# Exploring and enhancing the accessibility of children's oral health resources (called HABIT) for high risk communities

**DOI:** 10.3389/froh.2024.1392388

**Published:** 2024-11-04

**Authors:** Amrit Chauhan, Annalea Staples, Eleanor Forshaw, Timothy Zoltie, Riffat Nasser, Kara A. Gray-Burrows, Peter F. Day

**Affiliations:** ^1^ School of Dentistry, University of Leeds, Leeds, United Kingdom; ^2^ Better Start Bradford, Bradford, United Kingdom; ^3^ Community Dental Service, Bradford District Care NHS Foundation Trust, Bradford, United Kingdom

**Keywords:** intervention, oral health advice, limited English proficiency (LEP), oral health accessibility, dental caries, prevention, early years, community engagement

## Abstract

**Background:**

Within the city of Bradford in West Yorkshire, South Asian and Eastern European communities have an increased risk of childhood tooth decay, especially among families with Limited English Proficiency. Tooth decay is preventable, with national guidelines advocating home-based behaviours (toothbrushing with fluoride toothpaste and reducing sugar intake). In England, Health Visitors have opportunities to undertake oral health conversations during universal visits for children aged 0–24 months. The HABIT (Health visitors delivering Advice in Britain on Infant Toothbrushing) intervention provides structured oral health conversations, underpinned by complex intervention methodology. A feasibility study found HABIT acceptable to parents, feasible to deliver and led to improvements in home-based behaviours. However, the reach of this original study was limited to those proficient in English. This new study focused on exploring and enhancing the accessibility of the HABIT intervention to parents with Limited English Proficiency.

**Method:**

Twenty-four parents participated in interviews and focus groups, with 21 requesting support from interpreters. Community centres and WhatsApp were used to maximise inclusivity. Interviews and focus groups, followed a topic guide and the “Think Aloud” technique, were professionally transcribed, managed in NVivo, and thematically analysed. Team discussions facilitated analytical rigour. Recruitment continued until data saturation.

**Results:**

Three themes were developed: (1) Navigating linguistic barriers; (2) Engagement through visuals; and (3) Addressing oral health challenges. Parents employed diverse strategies to interpret resources, including Google Translate, as well as family and wider community members. Consequently, the HABIT resources were modified to include simple text, subtitles, and translation tools. Parents highlighted the benefits of shorter oral health messages with clear visuals to help understanding, and this strategy was applied across all resources. Challenges surrounding children's resistance to toothbrushing, high sugar intake within their wider families and communities, and limited dental access were all raised. The HABIT resources were updated to address these challenges.

**Conclusion:**

Collaborative community engagement has enhanced the HABIT resources, enabling access for high-risk communities to preventive oral-health programmes thereby promoting health equity.

## 1 Introduction

Dental caries is one of the most prevalent childhood diseases globally, and a major public health priority (1, 2). In England, by the age of five, approximately a quarter of children are affected by dental caries (3). There are significant variations seen in both the prevalence and severity of dental caries; in the least deprived areas of England the prevalence of dental caries is 13.7%, with this figure rising to over a third at 36% in Bradford, a city in West Yorkshire with some of the most deprived areas across the country (4).

Dental caries is, however, preventable, with national guidelines identifying strong evidence for key oral health behaviours such as brushing twice daily with a fluoridated toothpaste and limiting the intake of sugary foods and drinks (5). Whilst many parents are aware of these behaviours, there is a research gap surrounding how to support families from high-risk communities to undertake these optimal oral health behaviours at home.

This current paper sits alongside a wider research project known collectively as HABIT^1^ (Health Visitors delivering Advice in Britain on Infant Toothbrushing). HABIT is an oral health intervention that supports Health Visiting teams to have effective oral health conversations with parents of young children. Co-designed with parents and health visiting teams in Bradford, HABIT is underpinned by robust behaviour change theory and has been formally tested in a Medical Research Council funded feasibility study (6). This found HABIT to be acceptable to parents, feasible to be delivered by Health Visiting teams and led to improved oral health behaviours over a three-month period (7). The study protocol outlines the key components of the intervention, including the development of parent facing resources designed to support the highly valuable oral health conversations taking place between Health Visiting teams and parents of young children (8). These resources are an example of good practice and include a website, printable leaflets, dental models for toothbrushing demonstrations and six educational videos.

While the HABIT intervention demonstrates promising outcomes in enhancing oral health awareness and practices among parents, one of the limitations of the feasibility study was the lack of participation from parents with Limited English Proficiency (LEP) (7). With disparities in health outcomes persisting, marginalised populations (including those from minority ethnic communities) often face greater challenges in accessing essential oral health information and services (9). Just under half of Bradford's population identifies as within a Minority Ethnic group, with the Asian or British Asian population accounting for 32.1% of the district's total in 2021 (10). The challenges that these communities face in accessing healthcare are well documented (11, 12), with language barriers specifically leading to reduced comprehension of health information (13), increased likelihood of medical errors, delayed or inadequate care, and lower levels of patient satisfaction (14).

After the initial stages of this project, further funding was secured from Better Start Bradford's^2^ Innovation Fund. A key objective of this research was to work with local communities at high risk of early childhood caries to ensure accessibility of the HABIT resources for parents with LEP.

### 1.1 Aim

To explore and enhance the accessibility of the HABIT resources for parents/guardians with LEP.

## 2 Materials and methods

### 2.1 Research design

The research employed an exploratory qualitative study design, with reporting guided by the Consolidated Criteria for Reporting Qualitative Research (COREQ). Data was collected using in-depth interviews and focus groups and analysed using Thematic Analysis at the semantic level. This approach focuses on identifying explicit and surface-level meanings of the data, interpreting data in a way that stays close to the participants' stated experiences and perspectives (15). Ethical approval was obtained for the study from the University of Leeds Dental Research Ethics Committee. Ref: 180620/PD/301.

### 2.2 Sample

In collaboration with Bradford Community Dental Service and the funder (Better Start Bradford), two communities with a high prevalence of dental caries in young children were identified: South Asian and Eastern European. Participants were eligible if they were: (1) living within the Better Start Bradford area (Bowling and Barkerend, Bradford Moor and Little Horton), (2) had caring responsibilities for at least one child aged 0–4 years old, and (3) had LEP or English as a second language.

Participants who met the eligibility criteria were approached for participation by community workers familiar to them. The community workers shared the participant information sheet and subsequently obtained written consent. For those unable to read English, community workers provided translation assistance for both the information sheet and the consent form. Participants were given the option of attending their local community setting or via WhatsApp video/voice call to undertake the subsequent interviews/focus groups.

For interviews conducted via WhatsApp video or phone, participants signed the consent forms, and they (or their community worker) sent a photograph of the signed document back to the researchers undertaking this study. All participants were informed about the nature of the research, emphasising that their involvement was voluntary. They were also made aware of their right to withdraw consent up until the audio transcription stage. Recruitment continued until data saturation occurred. When arranging the interviews/focus groups, participants were asked if they wanted the community worker to help to interpret the discussions.

### 2.3 Data collection

Interviews and focus groups were undertaken by two female researchers (AC and AS) from different disciplines (Psychology and Dentistry). AC is an experienced qualitative researcher (CPsychol, PhD, BSc), and AS is a dental therapist and researcher (Grad Dip DHDT, PG Cert in Health Research). The researchers were unknown to the participants.

Community workers participated in discussions with the research team before the interviews to clarify their role of strictly translating the communication between the researcher and participant. Although the community workers did not receive formal training, their extensive experience in community engagement and their work with Better Start Bradford ensured expertise. The community workers participated in debrief sessions with AC after each interview to reflect on their experiences and contribute to the iterative refinement of the interview approach.

#### 2.3.1 Individual interviews

Interviews were carried out through the online instant messaging service WhatsApp video, a secure app that uses end-to-end encryption or by telephone and lasted between 30 and 45 min. These interviews were arranged by community workers through Better Start Bradford, and these workers were virtually present for four interviews. The researcher (AC) initiated the three-way video call, ensuring all were present and the video and audio connections were clear. Three interviewees felt they were able to engage in a conversation without the support of a community worker, and therefore, a two-way video interview was conducted.

#### 2.3.2 Focus groups

The focus groups were undertaken by two researchers (AC and AS) in various community settings, including community centres, primary schools and at English for Speakers of Other Languages (ESOL) classes. These sessions lasted between 30 and 60 min. A community worker was present for all focus groups (*N* = 4) to interpret discussions.

All interviews/focus groups followed a semi-structured topic guide (see Supplementary File). This included (1) an exploration of current children's toothbrushing practices within the community, and (2) an exploration of the parent-facing HABIT oral health resources. The researchers initially posed the interview questions in English to the participants, and where the interviewee was not fluent in English or required additional support, the community worker translated the researcher's questions into the appropriate language in real-time. The interviewee's responses were then translated back into English by the community worker. The participants were encouraged to employ the “Think Aloud” technique (16). Guided by the topic guide, this allowed participants to verbalise their thought processes in real time, thereby gaining deeper insights into their perspectives and understanding of the subject matter. Following each interview or focus group, the researchers conducted field notes and debriefed.

To ensure an iterative process, the HABIT resources were edited and improved in real time. Any adjustments were then highlighted within the following set of interviews and focus groups for further feedback and revision suggestions.

### 2.4 Analysis

Focus groups and interviews were recorded, and the English content of the discussions was professionally transcribed verbatim. Transcripts were checked and anonymised before the data was analysed at a semantic level using the following steps of Thematic Analysis (15):

1. Familiarisation
2. Coding
3. Searching for themes
4. Review themes
5. Defining and naming themes
6. Producing the report

During familiarisation (phase 1), the data was actively read in search of meanings and patterns before coding using NVivo (phase 2). The coding of the initial interviews was undertaken by AC, and as the study progressed to focus groups, the second researcher (AS) joined, contributing to the refinement and coding of the subsequent interviews and focus groups. The next phase involved refocusing on a broader level at “themes” rather than individual “codes” (phase 3) before reviewing (phase 4). Each theme was then refined and explicitly named, aiming to capture the essence of each theme (phase 5). Theme identification was derived solely from the data and was undertaken by AS under the supervision of AC. Each phase was reviewed by both researchers to facilitate analytical rigour, to ensure consistency and to discuss emerging themes, before collaborating for the final defining and naming of themes in phase 5.

## 3 Results

### 3.1 Overview

In total, 24 individuals took part in the study, and reasons for non-participation were not sought. Further demographics are provided in Table 1. Interviews with parents (*N* = 7) took place between February 2021 and June 2021, during a peak wave of the COVID-19 pandemic, by one researcher (AC). A total of four focus groups with parents (*N* = 17) took place between November 2021 and December 2022 and lasted between (20–35 min). Of those focus groups, there were two groups of 5 parents, one group of 3 parents and one final group of 4 parents.

**Table 1:** Participant demographics.

Ethnicity	Total participants	Those requiring interpreting services	Gender		Familial role		
			Male	Female	Mother	Father	Grandparent
South Asian	12	8	1	11	11	1	0
Eastern European	12	12	3	9	8	3	1

In evaluating the broader accessibility of the HABIT resources, three main themes were developed (1) Navigating linguistic barriers, (2) Parental engagement through visuals, and (3) Addressing oral health challenges. The themes and subthemes can be found below in Table 2. How the themes from the thematic analysis directly informed the enhancements of the HABIT resources are then outlined. For consistency and ease of reference in this paper, all participants will be collectively referred to as “parents”.

**Table 2:** Themes and subthemes.

Theme	Subthemes
1. Navigating linguistic barriers	1a. Use of Google Translate to interpret HABIT resources
	1b. Seeking assistance from family and wider community members
	1c. The role of visuals to help interpretation
2. Parental engagement through visuals	2a. The role of visuals to help to reinforce key HABIT messages
	2b. Resources need to be short and concise
	2c. The resources should be visually pleasing
3. Addressing oral health challenges	3a. Children being resistant to toothbrushing
	3b. High sugar consumption within the wider family and community
	3c. Difficulty accessing a dentist

### 3.2 Theme one: navigating linguistic barriers

Parents employed diverse strategies to interpret the HABIT resources, including Google Translate, help from family and wider community members and visual aids. These strategies underscore the critical need for HABIT resources to be available in an individual's first language, ensuring better understanding of the content and messages conveyed. As one interpreter explained:

*Interpreter (I): “She'd [i005] only really understand it if it was in her language.”*

#### 3.2.1 Use of google translate to interpret HABIT resources

One prominent sub-theme was the use of digital interpretation tools. Parents frequently described how they used Google Translate as a primary method for interpreting the HABIT resources. This was a necessity due to the language barriers:

*Parent (P): Yes, be better maybe in Romanian language maybe, it is better…it's normal use translate.*

*I: Okay, so is that how you normally do it, you put it in Google Translate. That's really good…do you all have the access?*

*P: Yeah, every single person got the access to the [Google Translate]” - Focus Group 25.10.21*

**Figure 1:**
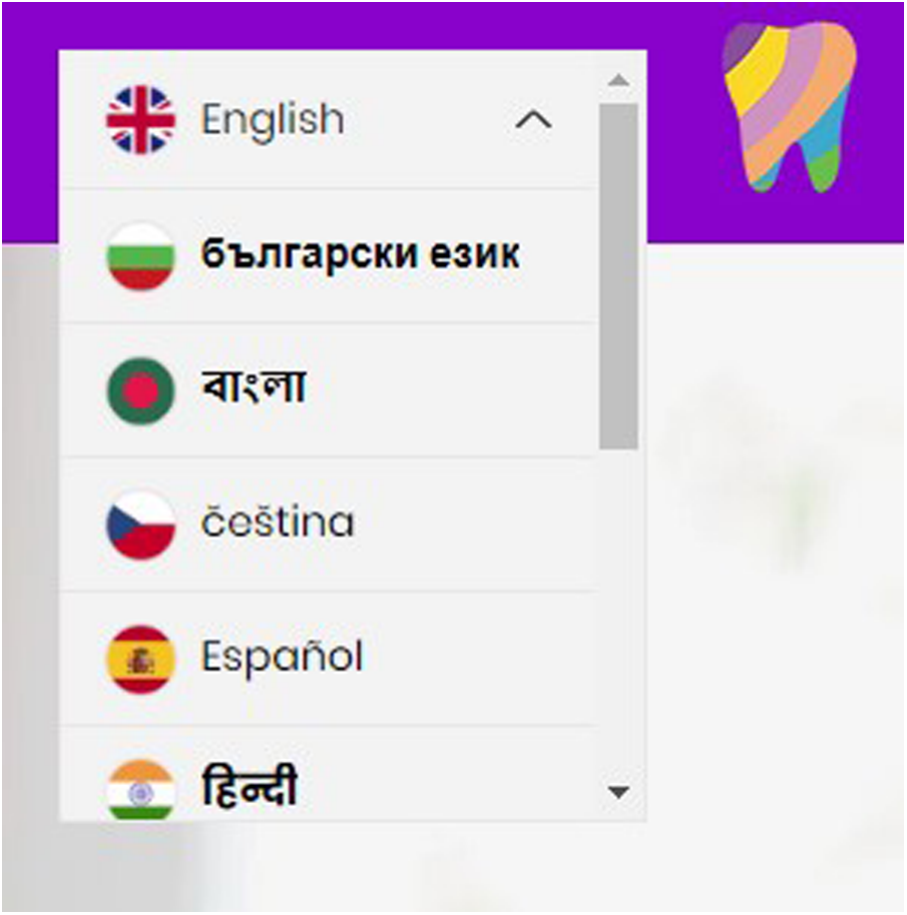
Embedded translation tool within the HABIT website.

The parent's response, “it's normal use translate”, indicates Google Translate is a common and accepted practice, where “every single person got the access to the [Google Translate]” suggests the widespread availability and accessibility of this tool within their community. The need to use Google Translate highlights a gap in the HABIT resources, specifically the absence of built-in language translation features within the website. Subsequently, the HABIT website incorporated a translation feature, aiming to reduce dependence on Google Translate and help understand the content of the website (see Figure 1).

**Figure 2:**
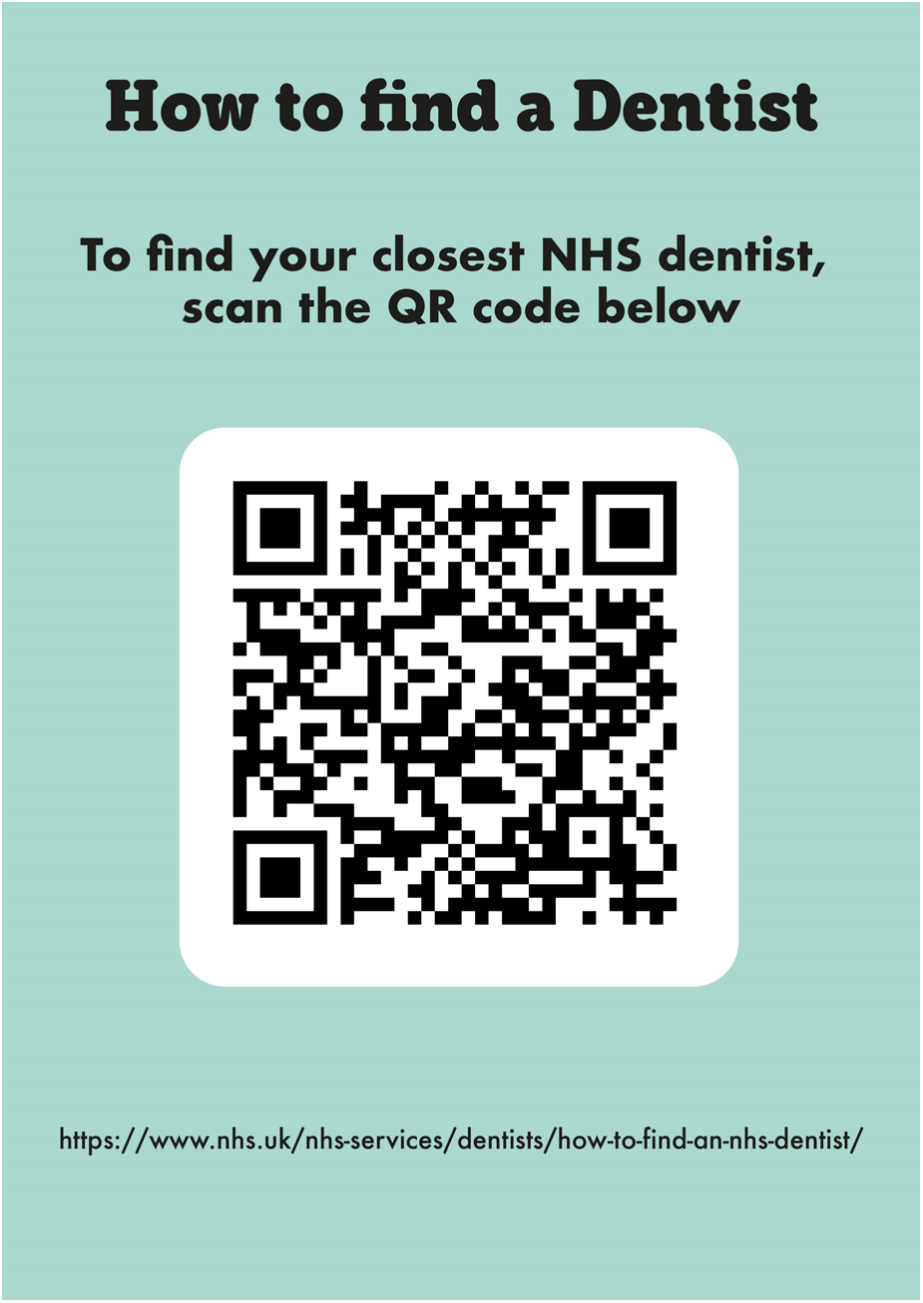
A screenshot of google translate on the HABIT leaflet.

For the leaflets, a specific section has been added to encourage parents to use Google Translate. For the videos, a translation feature has been embedded. This allows parents to access the content in their preferred language using YouTube's translation capabilities. This integrated approach across the website, videos and leaflets ensured language barriers were minimised and acknowledges the community's widespread use of the Google Translate App (see Figure 2).

#### 3.2.2 Seeking assistance from family and wider community members

Another subtheme highlighted how parents with LEP may seek assistance from family members, friends, or community members to translate the HABIT resources.

Some participants highlighted broader health barriers, particularly when understanding written communication from a wider range of healthcare professionals, such as GPs. One parent shared an example of this:

*P: Because when it's like the [GP] letter…anything I can't understand, I ask some people here, my children or my friends who understand English, they can explain me everything, yeah. No, I have support people here, they can tell me anything I can't understand, so that is no problem, yeah. - i003*

This narrative reflects the reliance on informal support networks to translate health resources and demonstrates the roles within families and the wider community. It is particularly interesting to note how children often became important in this translation process, with children supporting their parents in navigating language barriers. Parents similarly reported their experiences with community members for translation assistance:

*I: “Some can understand even they just see the video, but I can translate it, but it's okay in the video because video we can see everything.” - i003*

**Figure 3:**
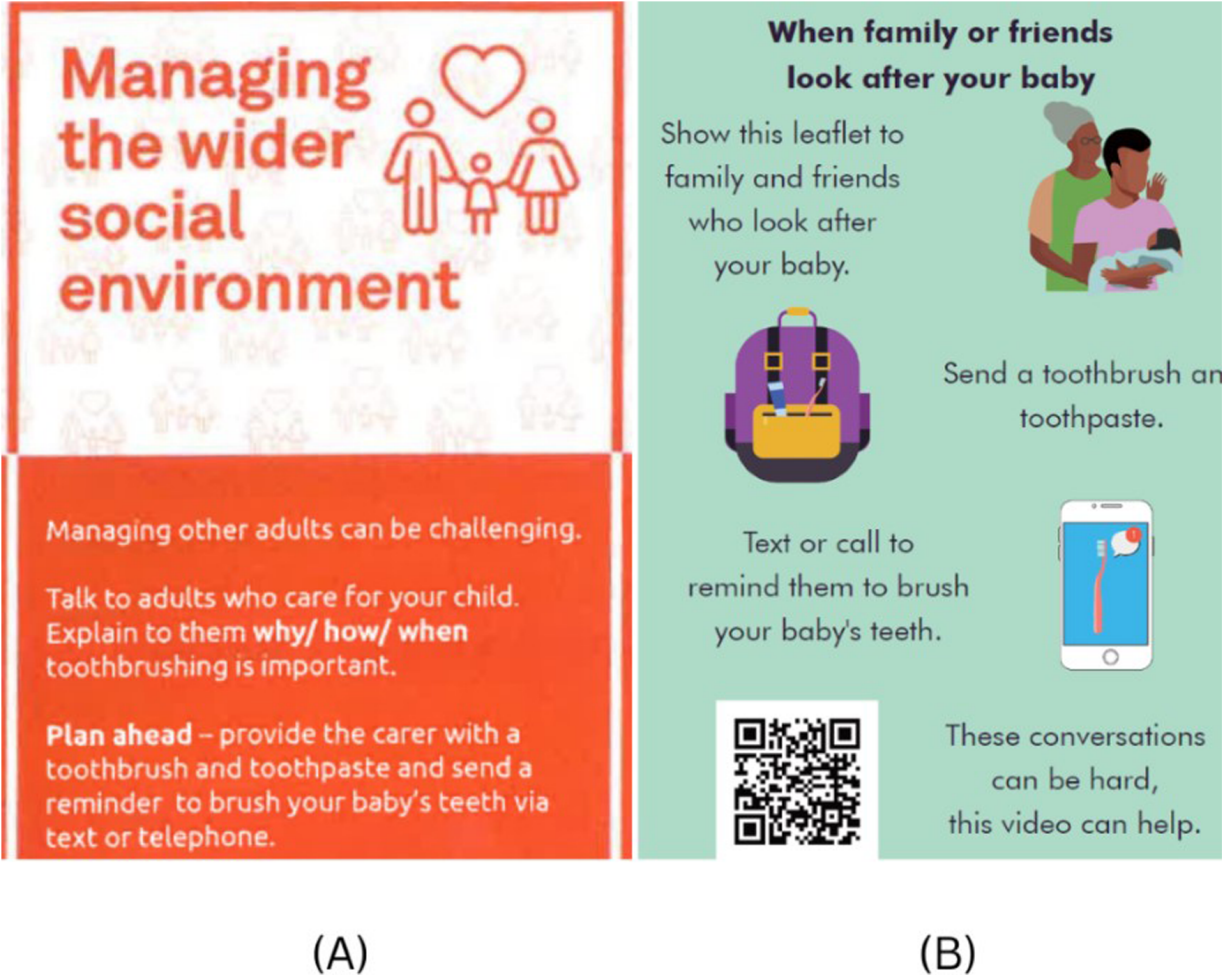
Example screenshot of both the previous **(A)** and recent **(B)** versions of the HABIT leaflets.

Both highlight how support networks can help overcome language barriers. The support from younger family members, particularly, highlights the need for HABIT to be accessible and understandable across different age groups and language abilities. This included simple language choices and avoiding complex dental terminology. Figure 3 demonstrates how the language in the HABIT resources was simplified to be clearer and more concise. This, in turn could facilitate easier translation, particularly by younger family members who may play a crucial role in interpreting the HABIT resources for parents. The leaflet was evaluated utilising the widely used Gunning FOG (Frequency of Gobbledygook) index to ensure that the resources reflected an appropriate readability level for the target audience, with a final score of 5.08 indicating accessibility to those with a reading age of below 11 years old (17).

#### 3.2.3 The role of visuals to help interpretation

This sub-theme highlights the significant role that visual aids (including images and videos) had in interpreting the HABIT resources, particularly in overcoming language barriers. Parents specifically pointed out that when textual content within the HABIT resources posed challenges in translation or interpretation, visual components helped with their understanding:

*P: “For one example, looking the video, no need speaking. Because you understand without talk. Image explain everything.” - Focus Group 25.10.21*

The narrative suggests that visuals, particularly the video resources within HABIT, helped communicate oral health messages in a universally understandable way. The parent's narrative that “image explains everything” highlights how visual aids can provide clear information, reducing the reliance on text-based explanations and therefore linguistic barriers. As such, further enhancements were made to ensure that the visual components were not only clear, but also directly aligned with the spoken language. For example, instead of displaying a range of drinks the visuals now illustrate just water and milk when discussing drinks that are safer for the teeth.

### 3.3 Theme two: parental engagement through visuals

It became evident that visual aids were not only essential for aiding interpretation but could also influence parents’ willingness to engage with the resources. A key aspect was how these visuals were presented, including the length and complexity of visual materials and their overall aesthetic look.

#### 3.3.1 The role of visuals to help to reinforce key HABIT messages

Parents for example, suggested that visual aids could help reinforce the key messages of the HABIT resources:

*P: “you know, like in some, usually they have those diagrams in dentists and stuff, like sugar, and have like of course not to eat, like basically telling you not to eat that and that's bad for your teeth, so like, what's good for your teeth and what's bad for your teeth”- i002*

The parent's reference to diagrams commonly seen in dental clinics demonstrates how parents may favour clear, illustrative diagrams. Their recollection of these specific diagrams indicates not only an understanding of the content but also their engagement with the dental resource. The parent remembering these visuals from a dental setting suggests that such diagrams effectively capture attention and reinforce oral health messages. This was similarly shared with another participant when discussing the HABIT videos:

*P: “It's okay in the video because video we can see everything because they show in the video, it's very good, because they show us what is good and what is bad. Which is the health food and which is the bad food, no.”- i003*

**Figure 4:**
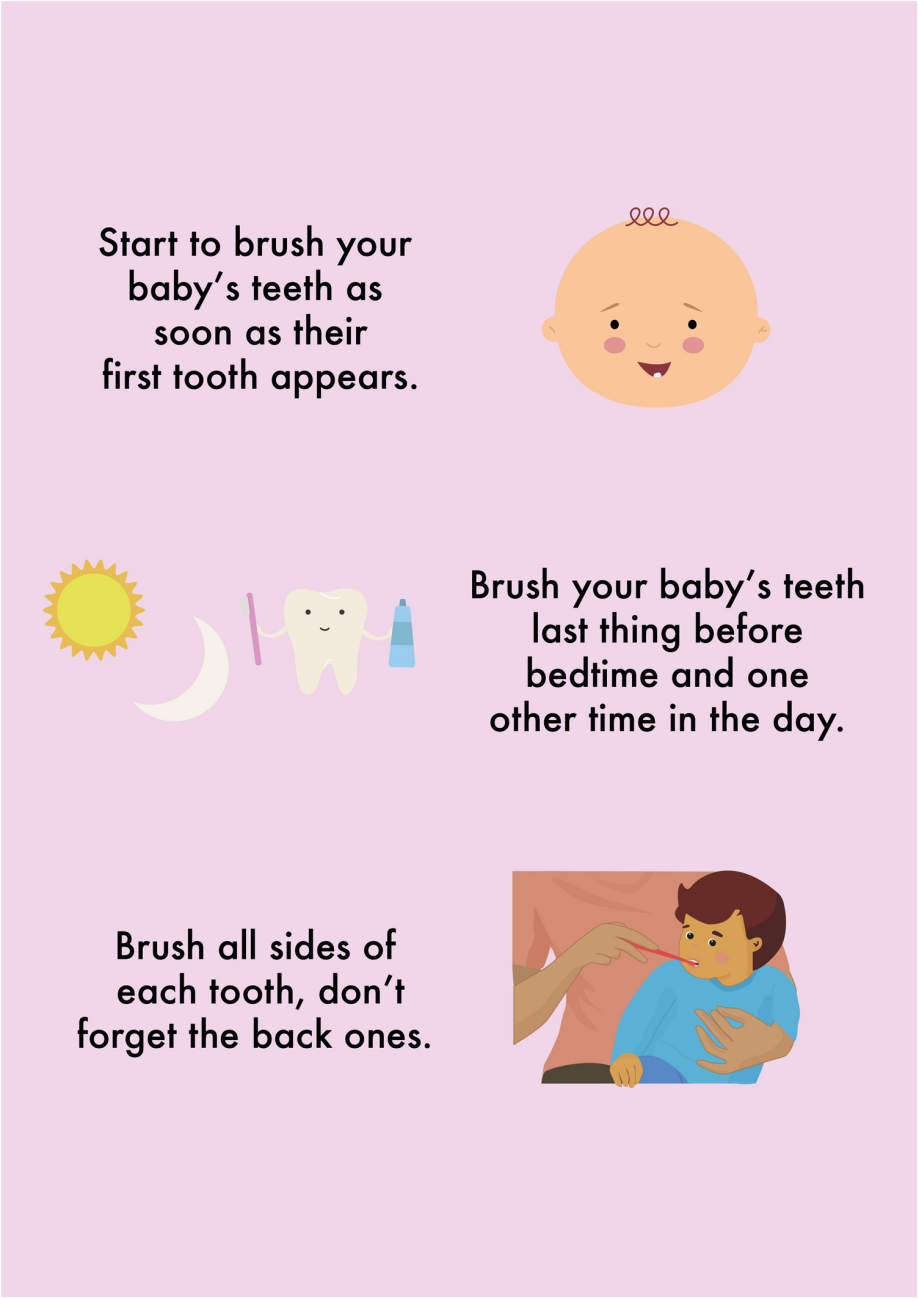
Example of HABIT leaflet “Brushing your baby's teeth”.

This parent's preference for the video format indicates its potential as an engaging and informative tool. The positive responses to these formats meant that the HABIT resources were further refined to ensure that the videos were easy to access and that the QR code linked to the video was included on the leaflet. Furthermore, the leaflet was further enhanced to include diagrams and visuals alongside key oral health messages. Figure 4 showcases part of the updated HABIT leaflet, where a corresponding visual aid accompanies each key message. For instance, the message “only use a smear of toothpaste” is visually represented by an image of a toothbrush with the correct amount of toothpaste. This imagery is illustrative and instructive, providing a clear image of the recommended amount. Such visuals contribute to the overall appeal of the leaflet, making it more engaging for the parents to review and follow.

This not only captures immediate attention but could also help to reinforce vital oral health messages, making them more accessible and memorable for parents.

#### 3.3.2 Resources need to be short and concise

Another aspect of the HABIT resources, as highlighted by parents, was the need for short and concise information, especially within the HABIT videos:

*P: “I think four minutes too long, because basically, the message in it and it's, you can tell by straight away, the first two. It's nice to hear the people, but it's the same thing innit, the importance of brushing the teeth, the effects long-term” - i007*

The parent perceived the initial part of the video as sufficient for conveying the essential message, implying that prolonged repetition may lead to parents not watching the rest. While the participant valued hearing from other parents, the narrative highlights how these should be carefully integrated. As such, the HABIT resources were adapted to ensure that videos were shortened to an average of two minutes, focusing on delivering the key messages in an engaging format that succinctly conveys the key messages.

#### 3.3.3 The resources should be visually pleasing

As expressed by parents, an essential aspect of the HABIT resources was the need for these resources to be informative and visually engaging. This preference underscores the role of aesthetic appeal in capturing and maintaining interest. Through an interpreter, one participant provided valuable insights into this by comparing the HABIT leaflet with traditional, text-heavy GP letters:

*I: [i004] doesn't like the usual letter, it's just full of lines and, you know, you're not really interested because you can't understand it anyway, so if it's short and big words, like headlines, then it would be a lot easier for them to understand.*

For parents with LEP, lengthy documents filled with dense text could potentially be overwhelming and difficult to engage with. The participant's comment about preferring “short and big words, like headlines” suggests that breaking down information into smaller, easily digestible segments can significantly enhance understanding and engagement. Headlines or key points in larger fonts can help convey essential messages without overwhelming parents, a sentiment shared by another participant:

*I: “Firstly, she [i004] says pictures would make it easier, but then even writing, if you give a big heading, like in bold, and then give, like, tips underneath that, then it probably would be better.”*

**Figure 5:**
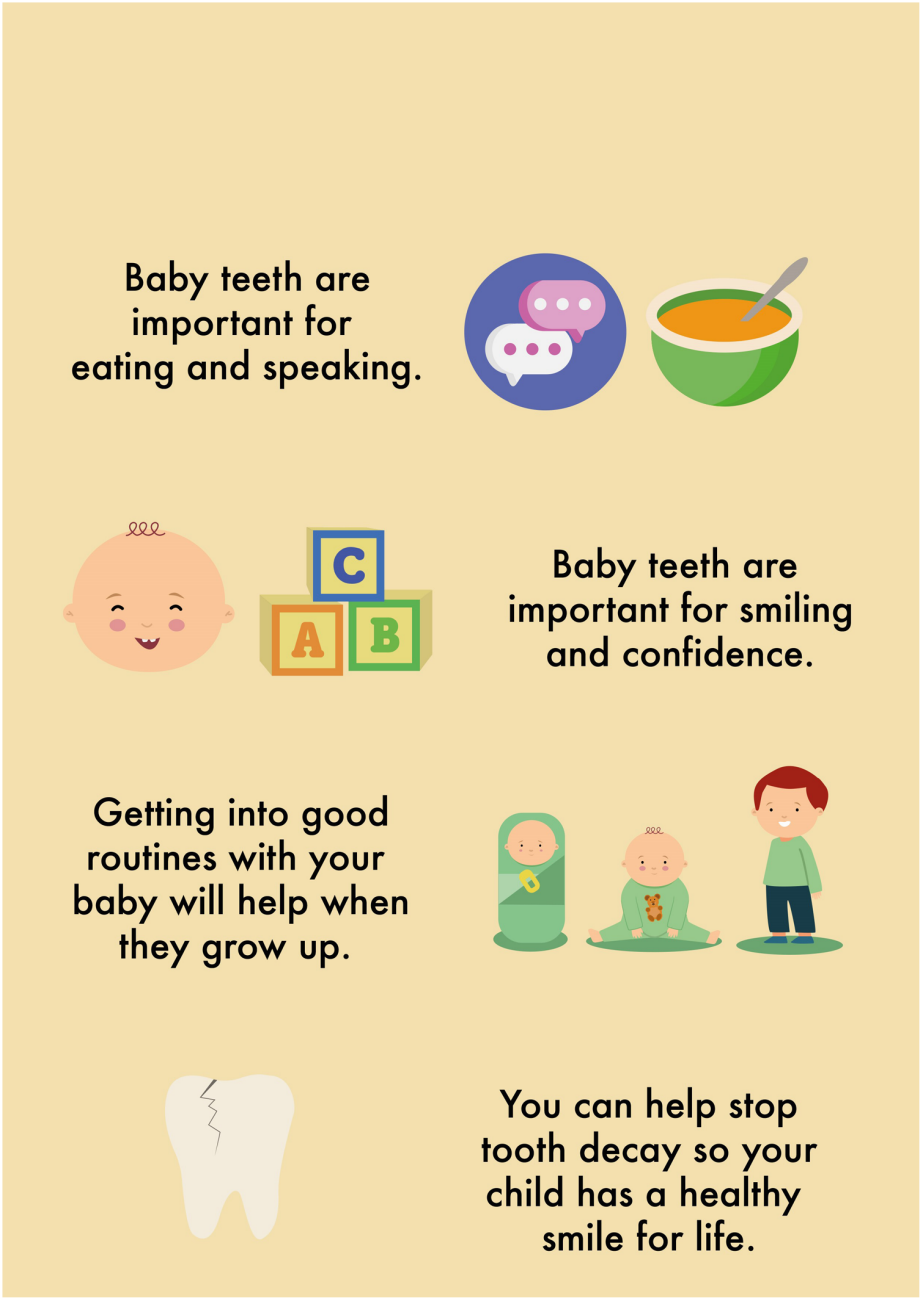
Images included on the “Your baby's teeth are important” section of the HABIT leaflet.

These recommendations highlight the importance of combining visual elements, like pictures, with clear and concise written content. This could potentially help prevent parents from becoming disinterested, as suggested by this participant. As such, the HABIT resources were further refined with key design elements that improve visual clarity and appeal (see Figure 5). This included key messages that were headlined with phrases such as “your baby's teeth are important” to draw attention and provide a quick, clear understanding of the topic. In addition to the textual changes, the visual aspects of the resources were also enhanced. The imagery and colour schemes were carefully selected and revised to make the resources more visually engaging.

### 3.4 Theme three: addressing oral health challenges

This theme captures the barriers experienced by parents in maintaining their children's oral health. These include difficulties in managing children's resistance to toothbrushing, navigating high sugar consumption within their wider family and community settings, and accessing dental care services.

#### 3.4.1 Children being resistant to toothbrushing

When discussing their experiences with toothbrushing, many parents in the study shared instances where their children showed a lack of interest or resistance to engage in toothbrushing:

*P: “morning for example, if I wake up now, she wouldn't wanna brush her teeth. She'll say to me she'll do it later or, and if I do try and encourage her, she'll just for a couple of seconds, she doesn't wanna brush it for longer.”- i002*

Interestingly, this participant's narrative suggests that toothbrushing is perceived as a task which can be postponed or negotiated rather than an essential part of their routine. The parent's attempt to “encourage her” to brush her teeth highlights the role of parents to motivate their children to develop good oral health habits. A significant challenge highlighted in this narrative, and shared by many, was ensuring that their children brushed their teeth for the recommended length of time. The participant further elaborated on this challenge and described how this became more difficult as the child grew older:

*P: “I could only go with my personal experience, you know, that kids sometimes they're reluctant to brush their teeth, or not brushing it long enough, because with my little one, at first she was really good, she was brushing from top to bottom, her back teeth, but now she just puts it in her mouth and she won't brush it for long, she doesn't like it now.”- i002*

This account illustrates the transition from a parent-led to a child-led toothbrushing routine. In the initial stages, the participant was actively involved in supervising the toothbrushing process, as evident from her description of her child brushing “from top to bottom, her back teeth”. As her child grew older, however, a shift was observed. The child's approach to toothbrushing changed as she became more independent but less thorough, which was shown by the child putting the toothbrush in her mouth and brushing for a shorter duration. While parents were aware of their roles in motivating or reminding their children, they also acknowledged that toothbrushing may not be done consistently or thoroughly. This was reflected by other participants:

*I: “She [i004] says to the kids to brush their teeth and they find it quite boring.”*

The refinement of the HABIT resources, informed by the findings from participant narratives, focused on two key aspects: the importance of parents brushing/assisting with their child's toothbrushing, and the need for strategies to make brushing more engaging for children. Firstly, the updated HABIT resources underscore the importance of continued active parental involvement in the toothbrushing process, even as children grow older and seek more independence. This is reflected in the recent update to the UK's Delivering Better Oral Health (DBOH) guidelines (5), where the use of the word “supervised”, which is relatively ambiguous, has been replaced with the more direct instruction for active participation; “adult involvement ensures the correct amount of toothpaste is used, enables them to prevent children eating or licking toothpaste from the tube and that all teeth are brushed thoroughly”. The HABIT resources have remained consistent with this messaging throughout. All videos and images used clearly displaying the parent actively brushing their child's teeth, and any related language making it evident that this level of engagement in brushing is necessary.

Secondly, the strategies to make toothbrushing a more positive and engaging activity for children have also been further refined, by incorporating interactive and educational content. This refinement was determined and developed based on participant feedback and insights gathered during the study. The website offers fun and informative play ideas for making oral hygiene and healthy eating more interesting for children and encourages the adoption of healthy habits in a way that is enjoyable for the whole family. These elements are also integrated throughout the leaflet and videos. Making the toothbrushing experience more enjoyable for children enables better support for parents and caregivers, thereby enhancing the overall effectiveness of the resources. This development process ensures that the HABIT resources are user-friendly and effective for these communities.

#### 3.4.2 High sugar consumption within wider family and community

Parents frequently discussed the challenge of managing their children's sugar intake within the broader context of family and community dynamics. Their narratives underscore the complexities and barriers encountered in managing the consumption of sugary foods and drinks. One participant highlights these challenges:

*P: “I'm not buying too much candy but my husband, when he go out anytime, he bring the candy and you know like, sweets, you know. So this is not, it's not nice, so he give the children, so that's why she have this problem.”- i003*

Despite one parent's efforts to maintain a healthier diet, her child's tooth decay was identified as a consequence of her husband's actions. Her expression, “it's not nice”, not only conveys her dissatisfaction but also underscores her recognition of the direct impact these sweets had on her child's dental health. This phrase, coupled with the frequency implied by “when he go out anytime”, heightened the sense of frustration and lack of control she felt. This conflict within the household reflects a broader challenge parents described, managing not only their practices but also navigating the differing approaches of other family members and cultural norms.

#### 3.4.3 Difficulty accessing a dentist

Parents encounter several barriers trying to access an NHS dentist. The narrative from one participant who faced difficulties in accessing affordable dental care in England, is indicative of the broader challenges encountered by parents in obtaining treatment:

*“I: Do you see the dentist here? [England]*

*P: No. Here is very expensive, you have to go Romania.*

*I: So you normally go to Romania to see the dentist?*

*P: Yeah.” - Focus Group 01.11.21*

This narrative highlights cost as a barrier to accessing dental care. Interestingly, this participant found it more feasible to travel to another country for dental services, underscoring the challenges some parents had finding affordable dental care locally.

Similarly, other parents described their difficulty in accessing dental care for their children:

*P: “One of the elder children have got a lot of problems with teeth and they need a dentist, they’re not getting the dentist.” – i006*

The narratives from the study highlight a gap in awareness among parents about the availability of free or lower-cost dental care options within the NHS. More specifically, parents are unsure how to access these services in urgent situations when a child is experiencing dental pain. Parents may not be fully informed about the NHS provisions that offer free dental care for children up to the age of 18 years, expectant mothers, and those who have given birth in the last 12 months.

**Figure 6:**
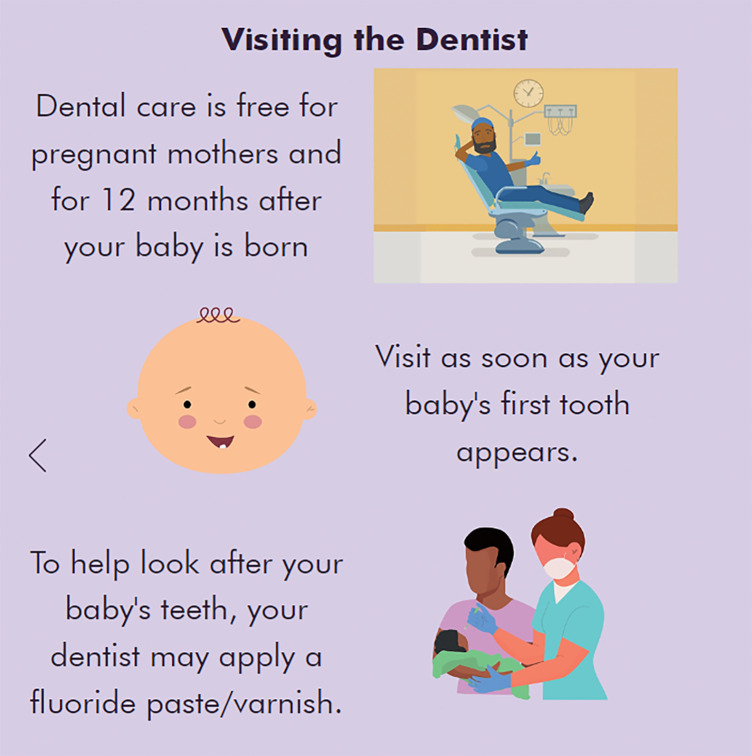
Signposting to dental services included within the HABIT leaflet.

The enhancements made to the HABIT resources were refined to reflect the identified needs and gaps in knowledge among parents with LEP. This included information about dental care being free (see Figure 6). This information is crucial as it clarifies dental access for expectant and new mothers, encouraging them to seek necessary dental care without the concern of cost barriers. The leaflet has therefore been updated to provide clear instructions on how to find a dentist, including information on accessing emergency dental services, particularly for children in pain (see Figure 6). Furthermore, a new video was created to specifically address the process of going to the dentist. This visual guide is designed to cover the key areas of concern for parents and to set expectations for what a visit to the dentist entails, making the information more accessible and engaging.

These findings underscore the difficulties that parents face while instilling good oral health habits in their children. Their actions and intentions can both be influenced by behavioural resistance, their broader social environments, and the systemic barriers to healthcare access that are often experienced.

## 4 Discussion

This study aimed to explore and enhance the accessibility of the HABIT resources for parents and guardians with LEP. The findings provided insights into the cultural, linguistic and health-related factors experienced by two communities identified as at a heightened risk of tooth decay (South Asian and Eastern European). It was clear that challenges surrounding engagement with the HABIT resources extended beyond language to the wider oral health barriers unique to each group, and these insights consequently played a crucial role in the refinement of the HABIT resources. These adaptations ensured that the intervention remained relevant and responsive to the evolving needs of the local population for which it was targeted. This was achieved through extensive community engagement, thematic analysis, and iterative modifications including simple text, translation tools, visual aids and shortened videos.

High sugar consumption was identified as one key barrier to oral health for both communities. For both South Asian and Eastern European communities, the high sugar consumption reported by parents is often not solely a dietary choice and can be deeply rooted in cultural norms and traditional customs (18, 19). The current study found this was often influenced by wider community members. Furthermore, Roma communities globally experience additional obstacles to healthy eating such as low incomes, lack of time, and difficulties in accessing appropriate preparation facilities (18, 20). It is evident that traditional health messages highlighting the impact of high sugar consumption (such as those seen in the initial version of HABIT), may not reach communities with LEP (21). This indicated a need to refine HABIT to be more culturally aligned with the unique traditions and experiences of these diverse communities, and calls attention to an area that wider health interventions need to be mindful of when addressing the broader barriers prevalent within a local context. By doing so, this approach ensures that health interventions are both effective and culturally sensitive, thereby enhancing their acceptability and impact within these specific population groups.

The current study also found that individuals with LEP used various methods to navigate and understand the HABIT resources. Similar to Pandey et al. (14), who explored immigrants with LEP experiences of healthcare access, the current study found that parents with LEP relied on language interpretations from their family or individuals within their community. Interestingly, however, our findings identified that children were frequently asked to interpret health resources. Pandey et al. (14) noted that the reliance on untrained interpreters such as family members or others from the community can lead to misinterpretation. The involvement of children in this role further heightens this risk, especially when traditional health resources may involve complex dental/medical terminology (22). Family members, however, may be the only available interpreters for those with LEP, and while non-professional interpretations may not be optimal, they are practical in lieu of other options for accessibility. Recognising this, enhancements were implemented in the HABIT resources specifically to support young interpreters, including simple language and readability checking. These modifications, which can be applied to broader health interventions, aim to ease the reliance on children and ensure that health messages are accurately conveyed to, and understood by, adult community members.

As professionals working within the community, Health Visitors are in a good position to help harness the collective experiences of vulnerable groups to identify and develop solutions that address their unique needs (23, 24), which can be extended to those with LEP. While translation apps like Google Translate were highlighted as useful tools in bridging language gaps (25), they do not replace the direct, meaningful conversations facilitated by Health Visitors. These professionals play a vital role in ensuring that health messages are not only accurately conveyed, but also understood in their intended context. The current study indicates that the integration of visually engaging resources, such as those enhanced for the HABIT intervention, is an important part in this process. Highlighting the importance of visuals to capture attention (through the use of colours), help with memory retention (through the use of imagery) and facilitate understanding (through the use videos), the study suggests that these types of resources could be a powerful supportive tool for Health Visitors to utilise when conveying oral health messages to those with LEP.

Recognising the involvement of family and wider community members for parents engaging with health information is also crucial, and in being aware of these dynamics Health Visitors can tailor their conversations more effectively. Within this context, testing interventions such as HABIT in parent and family-led activities could support the continued co-design of more effective resources. Parent-driven methods in peer groups can facilitate the exchange of both knowledge and support amongst wider family members and communities (26), which would encourage a more inclusive approach to oral health education. Leveraging community readiness in this way should contribute towards closing the current disparities in healthcare communication and accessibility, empowering community members by ensuring their voices and perspectives are integral in shaping the solutions that affect their lives.

In strengthening community action through active engagement, health practitioners can foster environments where health information is not only more accessible, but also more relevant to the specific needs of the population. This approach aligns with the principles of the Ottawa Charter (27) and emphasises the importance of participatory health promotion strategies. Critically, it underscores the significance of considering the socio-cultural contexts in which health behaviours occur, which should enhance the effectiveness of health interventions and ensure that they are both equitable and sustainable.

### 4.1 Future directions

Looking towards future enhancements of the HABIT intervention, an integrated approach across the early years services across the Bradford district will be a significant focus. Expanding the reach of the resources into community centres, family hubs, preschools and local clinics will increase its impact and ensure a more diverse audience, so with this in mind, the value of HABIT within other early-years settings is an area to be explored. Coordinating an integrative strategy will require consistent training for a range of professional disciplines and effective communication across the various stakeholders involved.

Longitudinal studies and service evaluations will also be required to assess the long-term impact of the accessible intervention on oral health behaviours and outcomes. The original feasibility study of HABIT reported improvements in plaque reduction and oral health behaviours. Tracking changes in oral health practices within vulnerable communities as a direct result of accessible interventions will provide further opportunities for evaluation.

### 4.2 Strengths and limitations

The inclusion of South Asian and Eastern European communities, typically underrepresented in oral health research, is a significant strength of this study, as these communities are at high risk of tooth decay (28). Working closely with neighbourhood engagement workers and community organisations, such as Better Start Bradford, significantly enhanced the recruitment and engagement process and led to meaningful adaptations in the research methodology. The use of WhatsApp as a communication tool was a suggestion from our partners, given they worked closely with these communities and were aware of their preferences. Future research should welcome the opportunities that similar collaborations can bring, as this can help to ensure communities are not overlooked in research and are given both a voice and an opportunity to participate. Overall, this works to ensure that the outcomes of the research are culturally relevant and prevent the widening of health inequalities.

Despite challenges posed by the COVID-19 pandemic which delayed recruitment, the study utilised WhatsApp videos to undertake interviews. This relatively novel approach in traditional qualitative research not only maintained communication throughout the pandemic, but also leveraged WhatsApp's widespread use throughout communities to enhance engagement with underserved groups. While this is considered a strength of the study, the limitations should be noted. Firstly, the use of WhatsApp meant that participants required a stable internet connection. Our discussions with research partners indicated that these communities frequently used WhatsApp, in preference to other digital platforms such as Zoom, Teams and Googlemeet. This meant that only individual interviews could be offered through WhatsApp as it is designed for individual communications or small group interactions and therefore impractical for larger focus groups. Once COVID-19 restrictions were lifted, alternative face-to-face methods were introduced. This shift aimed to accommodate those who might have been unable or unwilling to participate using this digital format. Recruiting participants through neighbourhood engagement workers and community organisations may also have limited participation to those who were actively engaged with the services and the activities Better Start Bradford provided.

Nevertheless, the use of inclusive methodologies like WhatsApp and collaborations with community networks played a significant role in enabling underrepresented communities to participate in oral health research. This approach demonstrates the value of adapting research methods to the preferences and needs of the community, thereby making studies more inclusive. By using both digital and face-to-face methods, and by working closely with community partners, the study was able to reach a broader cross-section of the community, enhancing the representativeness and inclusivity of its findings in the context of oral health.

## 5 Conclusion

Three key themes were identified when exploring the accessibility of the HABIT oral health resources for parents with LEP, and included navigating linguistic barriers, parental engagement through visuals, and addressing oral health challenges. The complexities of systemic barriers and cultural nuances were evident throughout and highlighted the impact of these experiences on the parent's overall access to oral health information and dental care. The findings derived from this study emphasised the importance of supplementing the existing HABIT oral health conversations between Health Visitors and parents with resources using an enhanced multimedia approach; prioritising culturally appropriate strategies that were easier to engage with for an audience with varying levels of English. Ultimately, this led to the significant refinement and improvement of the HABIT resources.

## Data Availability

The raw data supporting the conclusions of this article will be made available by the authors, without undue reservation.

## Footnotes

^1^ https://www.toothbrushinghabit.com/

^2^ https://www.betterstartbradford.org.uk/

## Author's note

PD and KG-B are the principal investigators for the grant and with co-applicants (AC, TZ and AS) oversaw the design and delivery of the research. EF and AC led the writing of the manuscript with major contributions from KG-B and PD. RF led the facilitation of the interviews and acted as a Project Oversight Manager.
