# Supplementary material for: Exploring and enhancing the accessibility of children's oral health resources (called HABIT) for high risk communities
Source: Front Oral Health. 2024 Nov 4;5:1392388. doi: 10.3389/froh.2024.1392388 (PMC11570590; doi:10.3389/froh.2024.1392388)
Supplement: Supplementary file 1 [file Supplementaryfile1.docx]

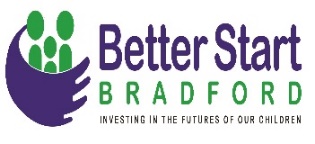

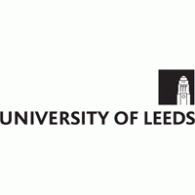


Views of the Habit (Health Visitors Delivering Advice in Britain on Infant Toothbrushing) Intervention

# Parents’ interview guide

[Information about the accessibility of the resources have followed the European standards for making information easy to read and understand: [file:///C:/Users/denabh/Downloads/Information_for_all_Inclusion%20Europe.pdf](file:///C:\Users\denabh\Downloads\Information_for_all_Inclusion%20Europe.pdf)]

## Before the focus group starts:

- Check that the 2 recorders have new batteries and are working properly before going into the FG.
- Specify how long the FG will take.
- Bring extra copies of information sheets and consent forms.
- Make sure all consent forms are signed.
- Inform about the bathroom location

## Introductions

- Thank you for taking the time to attend this discussion
- The purpose of today's focus group is to find out your views on some HABIT resources we developed about oral health which will be delivered by health visitors in your area.
- We would also like to know how this could be improved and how we can make the information reachable to other parents within your community.
- We work as a team of experts in different fields and this focus group will help us refine our develop these resources further so they can be applied effectively within the Bradford area
- There are no right or wrong views and opinions and I am aware that this is an area with diverse views and practices.
- But first of all, let’s start going round the table introducing ourselves. Name, how many children do you have, how old are they, do you take care after them on your own or does somebody help you, do you work. In other words, a little bit of a background, so we can get a better understanding. I’ll go first...

Stage 1: Exploration of current toothbrushing practices within the community

| - How important is your child’s oral health to you? - In your home setting, would you be the only one who takes care of baby’s oral health or is there anyone else, who would help you? What kind of help and support would it be? - Do you discuss oral health advice related issues with other parents within your community? What are the main similarities and differences, if any? |
| --- |
| - How familiar are you with how to take care children’s teeth? - Is this the same from others in your community? - Where do you get this knowledge from? - Is there anything you or others in your community would like to find out or to learn about? - Are there any areas of oral health you would like the Health Visitor to talk about (toothbrushing, diet, others)? - Is there anything you find difficult or hard? - Is there anything you find difficult or feel that some additional support, information or training would help you? What are these areas? |
| - In your opinion, what kind of resources should HV use in order to support you and other parents within your community and to improve children’s oral health? - If you already had an interaction with a HV, did he/ she use any resources? What were they and what impact did they have on your family? - If you haven’t met a Health Visitor yet, have you heard anything about how they deliver the support and advice and what kind of resources they use? - Are these helpful for those within your community? Why? |

Stage 2: Exploration of HABIT resources

Thank you very much for sharing your personal perspectives and experiences. We realise that some HV use different resources when they visit families, because of this we trained up HV within Bradford and gave them these resources to use when they visit parents at their 9-12 month visit. Now we would like to share these HABIT resources to gather some of your thoughts and find out what you think about them. Please take a few minutes to familiarise with the materials, explore, check and try them.

## Working in pairs Talk aloud initial thoughts.

Participants were asked to ‘think aloud’ as they view the HABIT leaflet and a section of the HABIT website in pairs, going around the focus group individually. The think aloud protocol is useful for evaluating how participants interpret and comprehend material. This will be the first time the resources will be introduced to gain their initial reaction.

Parents will be shown HABIT leaflet and a section of the HABIT website described above. For each resource, they were asked if they had seen the resources before and to think aloud as they looked at the image

- Tell me everything that you are thinking as you look at this leaflet/video.
- Tell me what you think this leaflet means, in your own words.
- What were you drawn to?
- What was you not drawn to?

Participants will also be asked to rate each picture from 1 (‘difficult to understand’) to 5 (‘easy to understand’) and the reasons for their response.

## Working in pairs to explore the HABIT resources

We would like you to work in pairs and think about where improvements might be made or service improved for all the resources (HABIT leaflet and website). If you think that we have missed anything and both of you think that there is something else that we have not presented to you, but you would use it in practice, please take the prepared sheet of paper and let us know about it.

- What was your favourite resource?
- Why did you choose these resources and not the others?
- How would they benefit those within your community?
- Is there anything you feel is missing to meet the needs of those within your community?
- Are there any other methods which can help people access these recourses within your community?
- Written information: These are things like leaflets, brochures and reports.
- Electronic information: This is written information on computers, for example information on websites or on CD-rom.
- Audio information: This is information you can listen to, for example on a CD or on the radio.
- Videos you can watch on TV tablet, mobile or on a computer
- Are the messages clear?
- What are your thoughts on the design and format of the resources? Eg. The size? The colours?

## Discussion on the HABIT leaflets

- Would you use this leaflet?
- If yes, how? If no, why?
- Will parents within your community be happy with the leaflet?
- Are the words easy to understand?
- Are the fonts clear and easy to read?
- Do you feel the information is ordered correctly?
- What are your thoughts on the images?
- Many people find it hard to read text- would they prefer images next to the information? photographs, drawings, or symbols?
- What are your thoughts on the action plan?
- Are these goals achievable?

## Discussion on the HABIT website

- Would you go onto this website?
- If yes, how? If no, why?
- Do those within your community have internet access?
- If not, what would be the best format for this information? For example, information on a CD or DVD may be better for some people than written information/ online information
- Would you know how to use them?
- What do you not like about the website/videos?
- Are the links easy to read and follow?
- Is there anything we can add to the website that will make the information easier for people to understand?

Discussion on the HABIT videos

- How can we make the videos more accessible to those within your community? (for example, the use of language, subtitles, translation or dubbing)
- What are your thoughts on the background voice of the videos, eg. are they slow and clear? Does the voice speak about things that people can see on the screen? Does it give enough time for people to understand the information? Is the accent ok?
- What are your thoughts about the length of the video?
- Some videos have a voice explaining what is happening in the film. This means that blind people can understand what is happening. Would this be useful to have?

## Concluding group discussion

- Would others within your community feel confident receiving these resources when receiving support from their health visitor?
- Are there any other barriers that may prevent you/others from using these resources?
- Is there anything else you would like to say that we have not asked?

## End

Thank you for your participation. We appreciate your time and a possibility to learn from your experience and expertise.

- Thank the respondents
- Remember to catalogue the tape/file
- During debriefing, reflect on, discuss with the research team and clearly note down any other issues (e.g. general impressions of the FG, questions that worked well, others that didn’t work) that may be important for future data collection, and data analysis.
